# Body mass index and serum markers associated with progression-free survival in lung cancer patients treated with immune checkpoint inhibitors

**DOI:** 10.1186/s12885-022-09744-5

**Published:** 2022-07-28

**Authors:** Zhenzhen Liu, Yuzhu Diao, Xiaoling Li

**Affiliations:** Department of Thoracic Cancer 1, Cancer Hospital of China Medical University, Liaoning Cancer Hospital & Institute, Xiaoheyan Road, Dadong District, Shenyang, 110042 Liaoning Province P. R. China

## Abstract

**Background:**

ICIs have remarkably affected the treatment strategies for numerous malignancies, including lung cancer. However, only a fraction of patients experience durable responses to ICIs; thus, there is an urgent need to identify the parameters related to ICI therapeutic effects. In this study, we investigated nutritional status surrogates and several serum markers to estimate the efficacy of ICIs.

**Materials and methods:**

The records of 66 patients with stage III/IV lung cancer who received ICIs were retrospectively analyzed. Features of patients’ clinical pathology, including age, sex, histology, line of treatment, BMI, serum albumin, serum creatinine, and serum inflammatory markers such as LMR and PLR, were examined. Progression-free survival was the primary endpoint. Relationships among categorical variables were assessed by the chi-squared test. Survival analysis was performed using the Kaplan–Meier method followed by the log-rank test. Cox multivariate analysis was performed to analyze the association between each variable and the survival time of patients.

**Results:**

The patients with BMI ≥ 25 (kg/m2), serum ALB≥37 (g/dL), serum creatinine ≥61.8 (μmol/L), LMR ≥ 2.12 had a significantly prolonged PFS in comparison with BMI<25 (kg/m2), ALB<37 (g/dL), creatinine<61.8 (μmol/L), LMR<2.12 (*p* < 0.05). No statistically significant difference was detected between patients with PLR < 135 and PLR ≥ 135 (*p* = 0.612). Multivariate analysis revealed that ALB≥37 (g/dL) and creatinine ≥ 61.8 (μmol/L) were associated with prolonged PFS, while statistical significance was not achieved in the BMI groups.

**Conclusions:**

The current results indicated that high BMI is related to longer PFS in lung cancer patients treated with ICIs, which may be correlated with high levels of serum albumin and creatinine.

## Introduction

Lung cancer is the world’s leading cause of cancer morbidity and mortality [1]. Immune checkpoint inhibitors, such as anti-PD-1/PD-L1 antibodies, have remarkably affected the therapeutic strategies for a variety of malignancies, including lung cancer, and could inhibit the tumor for a long time and even cure it [2, 3]. However, only a fraction of patients experience durable responses to ICIs, and there is an urgent need to identify the parameters related to ICI therapeutic effects.

To date, for the response to checkpoint inhibitors, the most researched predictive biomarkers include tumor mutation burden, PD-1 expression, CD4/CD8 lymphocyte ratio, the percentage of tumor-infiltrating lymphocytes, and several methods of establishing immune scores [4–6].

Recently, a few demographic features of patients have been studied to appraise the influence on ICIs [7]. One such feature, body mass index (BMI) or obesity, has attracted much attention. In addition, serum markers, such as albumin, creatinine, lymphocyte-to-monocyte ratio (LMR), and platelet-to-lymphocyte ratio (PLR), can be easily assessed because they can be measured from routine clinical laboratory tests.

Historically, BMI is regarded as the major substitute for nutritional status, but its association with clinical outcomes of patients with advanced stage tumors remains indeterminacy [8–11]. A meta-analysis indicated that a higher BMI level before treatment with ICIs was remarkably correlated with improvements in OS and PFS in tumor patients treated with ICIs, regardless of whether there were differences in the comparison models of BMI classifications [12]. However, one recent study showed that in 287 melanoma patients who received ICIs, BMI was not related to clinical benefit or toxicity [13].

The serum albumin (ALB) level is also often considered an indicator of patients’ nutritional condition in the clinical environment. Cachexia and sarcopenia are negative prognostic factors [14, 15]. Creatinine is usually used as a surrogate marker for sarcopenia; thus, we chose creatinine as a decisive variable. Using serum creatinine levels as an indirect assessment of skeletal muscle mass is an easy alternative when kidney function is accounted for [16–18]. The nutritional condition of patients might be influenced by the tumor microenvironment. The occurrence and progression of tumors are strongly associated with the inflammatory response, as inflammatory cells stimulate tumor cell multiplication and angiogenesis promotion, favor tumor invasion, and even affect the efficacy of some anticancer drugs [19].

In this retrospective study, we assessed the effect of BMI, ALB, serum creatinine and serum inflammatory markers such as LMR and PLR on survival outcomes in lung cancer patients receiving ICI treatments.

## Materials and methods

### Patient selection

The study was approved by the Ethics Committee of the Liaoning Cancer Institute and Hospital. This was a retrospective review of 66 patients with stage III/IV lung cancer between June 2018 and March 2021 in the Liaoning Cancer Hospital & Institute, the first ward of thoracic oncology, who received at least 1 dose of ICI therapy. The staging of lung cancer was based on the seventh or eighth edition TNM stage classification. Features of patients’ clinical pathology, including age, sex, histology, line of treatment, BMI (kg/m2), serum ALB (g/dL), serum creatinine (μmol/L), and serum inflammatory markers such as LMR and PLR, were examined. The above indicators were determined at the time of ICI therapy initiation. BMI was calculated according to the formula of weight/height2 (kilograms per square meter) and classified based on the WHO categories. For the purpose of this study, we used BMI </≥ 25 as the binomial cutoff. All patients were divided into two groups, nonoverweight (BMI < 25) and overweight/obese (BMI ≥ 25), for the final analysis. At the same time, we included underweight patients in the nonoverweight group. Progression-free survival (PFS) was determined by an investigator on the basis of a review of electronic medical records and defined as the time from treatment start to progression. RECIST V.1.1 was used to define objective response.

### Statistical analysis

Descriptive statistics or contingency tables were used to summarize the demographics and baseline characteristics of the patients. The cutoff value for LMR was 2.12 based on previous studies [20], and the cutoff value for PLR was 135 [21]. According to the peripheral laboratory reference range, the binomial cutoff for Alb </≥ 37 g/dL was used. The cutoff value for serum creatinine was 61.8 μmol/L (approximately equal to 0.7 mg/dl) [22]. Relationships among categorical variables were assessed by the chi-squared test. Survival analysis was performed by the Kaplan–Meier method followed by the log-rank test. Cox multivariate analysis was performed to analyze the association between each variable and the survival time of patients. *P* < 0.05 was considered statistically significant. All statistical analyses were carried out by SPSS v24.0 (SPSS, Inc.).

## Results

### Patient characteristics

Sixty-six patients with lung cancer were included in the research, and their clinical features are listed in Table 1. There were 19 women (28.8%) and 47 men (71.2%). There were 23 patients (34.8%) aged ≥65 years and 43 patients (65.2%) aged < 65 years. Fifty-two patients (78.8%) were treated with an anti-PD-1 agent, and 14 patients (21.2%) were treated with an anti-PD-L1 agent. ICIs were administered as first-line treatment in 29 patients (43.9%) and second or higher treatment in 37 patients (56.1%). According to the classification of histology type, non-small cells accounted for 77.3%, while small cells accounted for 22.7%. For the study purpose, 44 patients (66.7%) were divided into the nonoverweight group, and 22 patients (33.3%) were divided into the overweight/obese group. There were no significant differences between BMI, any serum marker group and age, sex, treatment line or immune checkpoint inhibitor type. However, a statistically significant difference was found between PLR and histology (*p* = 0.013).

**Table 1:** Characteristic of patients in this study (*N* = 66)

Characteristic	Total N (%)	BMI (kg/m2)			ALB (g/dL)			Creatinine (μmol/L)			LMR			PLR		
		< 25 (*n* = 44, 66.7%)	≥ 25 (*n* = 22, 33.3%)	*P*	< 37 (*n* = 13, 19.7%)	≥ 37 (*n* = 53, 80.3%)	*P*	< 61.8 (*n* = 37, 56.1%)	≥ 61.8 (*n* = 29, 43.9%)	*P*	< 2.12 (*n* = 12, 18.2%)	≥ 2.12 (*n* = 54, 81.8%)	*P*	< 135 (*n* = 22, 33.3%)	≥ 135 (*n* = 44, 66.7%)	*P*
**Age (years)**																
≥65	23 (34.8%)	15	8	0.855	7	16	0.109	12	11	0.642	4	19	0.903	6	17	0.361
<65	43 (65.2%)	29	14		6	37		25	18		8	35		16	27	
**Sex**																
Female	19 (28.8%)	12	7	0.701	3	16	0.612	14	5	0.067	3	16	0.749	6	13	0.848
Male	47 (71.2%)	32	15		10	37		23	24		9	38		16	31	
**Treatment line**																
First line	29 (43.9%)	20	9	0.726	6	23	0.858	14	15	0.259	6	23	0.64	7	22	0.161
Second or higher	37 (56.1%)	24	13		7	30		23	14		6	31		15	22	
**Histology**																
Non-small cell l	51 (77.3%)	33	18	0.533	12	39	0.149	29	22	0.809	10	41	0.58	21	30	0.013
Small cell	15 (22.7%)	11	4		1	14		8	7		2	13		1	14	
**ICIs**																
Anti-PD-1 agent	52 (78.8%)	33	19	0.287	11	41	0.566	30	22	0.607	10	42	0.67	20	32	0.089
Anti-PD-L1 agent	14 (21.2%)	11	3		2	12		7	7		2	12		2	12	

*ICIs* Immune checkpoint inhibitors, *BMI* body mass index, *ALB* serum albumin, *LMR* lymphocyte-to-monocyte ratio, *PLR* platelet-to-lymphocyte ratio

*P* values in bold indicate statistically significant differences (*P* < 0.05)

### Association of high and low BMI with PFS time

**Fig. 1:**
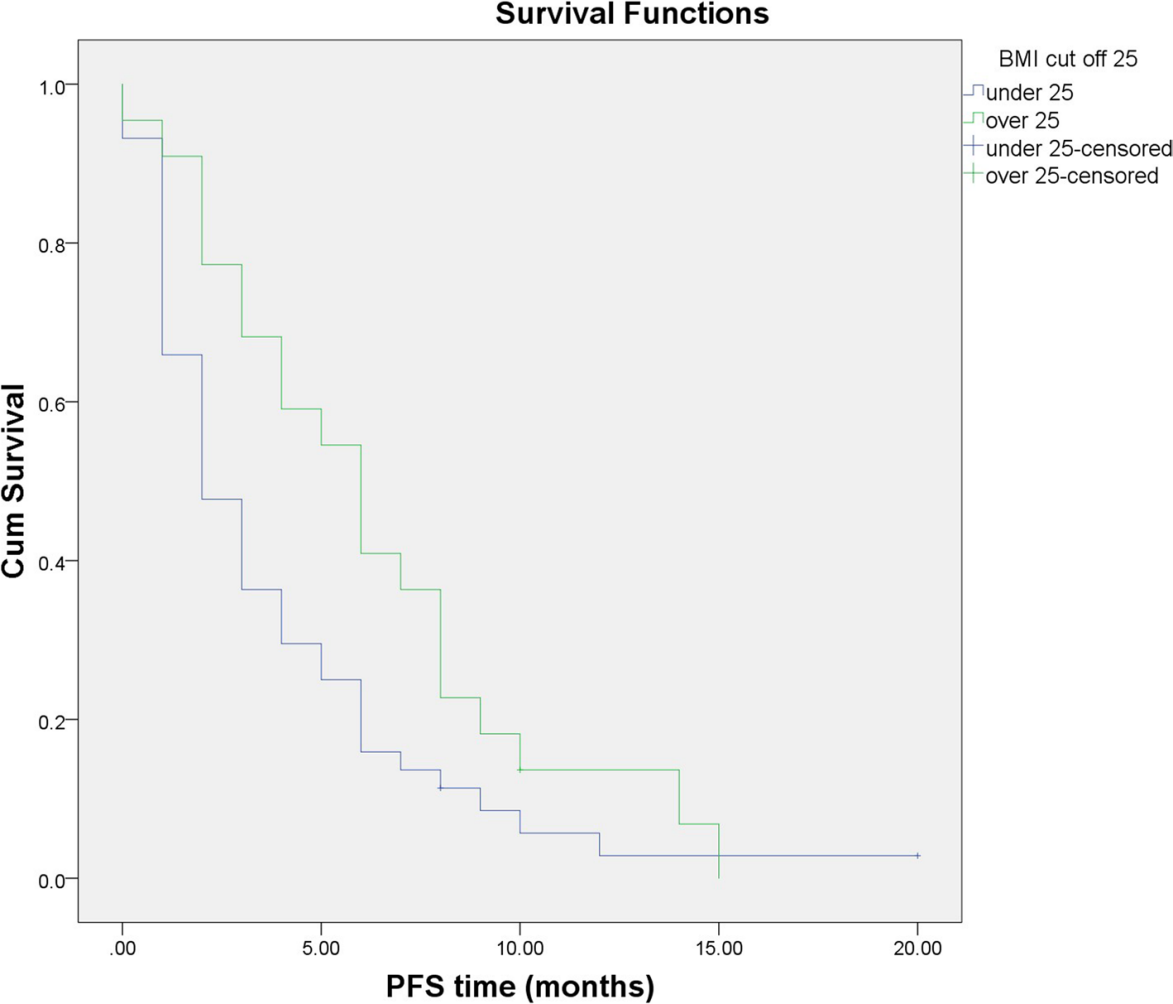
Progression-free survival in the nonoverweight and overweight/obese groups

In the study, all 66 patients were classified into nonoverweight and overweight/obese groups based on the BMI cutoff value. Statistical analysis of the PFS time of patients was performed for different BMI groups. The mean PFS times of the nonoverweight and overweight/obese groups were 3.81 months (95% CI 2.606–5.019 months) and 6.25 months (95% CI 4.446–8.054 months), respectively, and the difference was statistically significant (*p* = 0.04) (Fig. 1).

### Association of serum ALB with PFS time

**Fig. 2:**
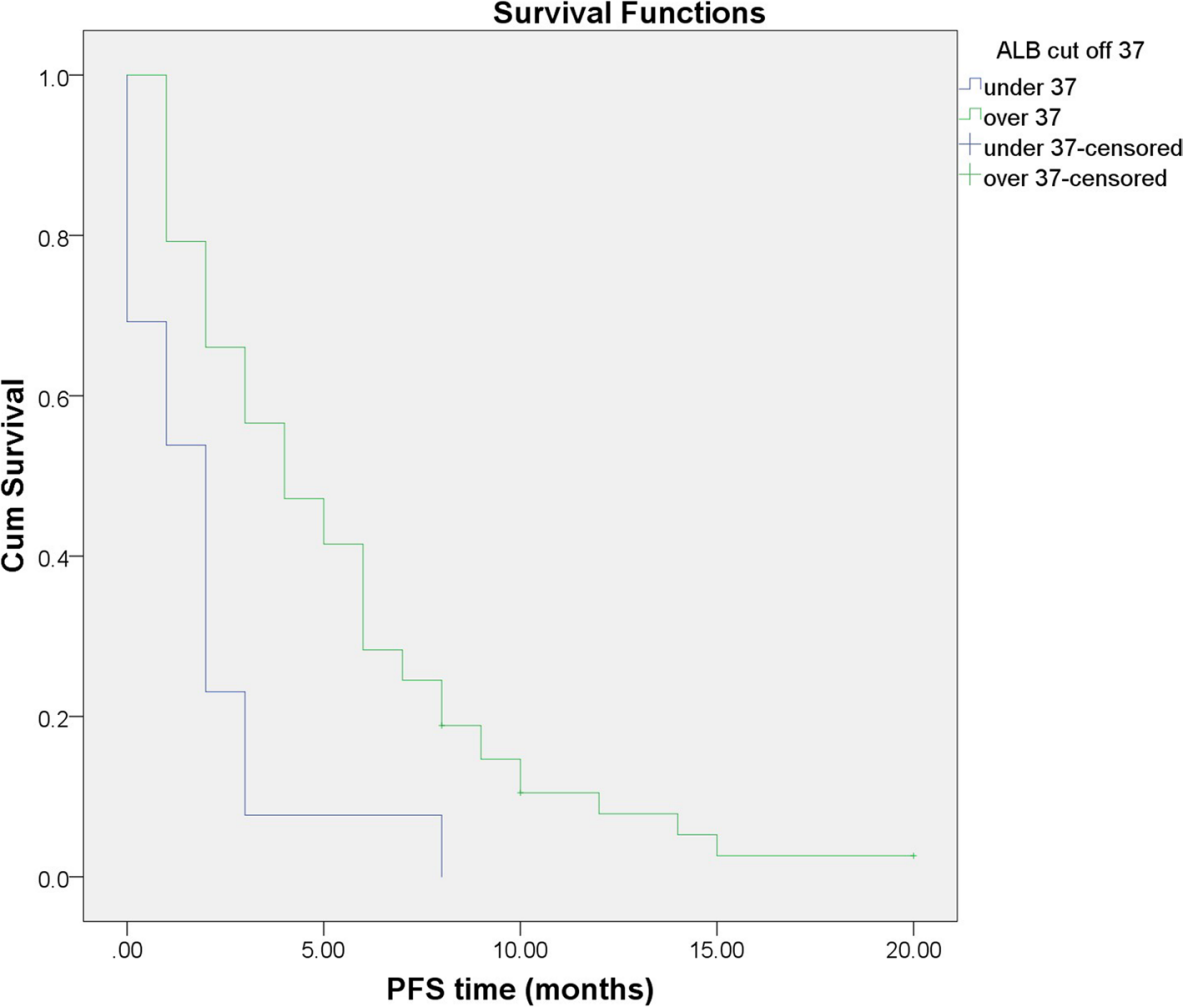
Progression-free survival in the high and low serum albumin (ALB) groups

Patients with baseline serum ALB≥37 g/dL had a longer PFS than those with ALB< 37 g/dL (the mean PFS was 5.32 months [95% CI: 4.120–6.519] vs. 1.85 months [95% CI: 0.675–3.017]), and we found that the difference was significant (*p* < 0.001) (Fig. 2).

### Association of serum creatinine with PFS time

**Fig. 3:**
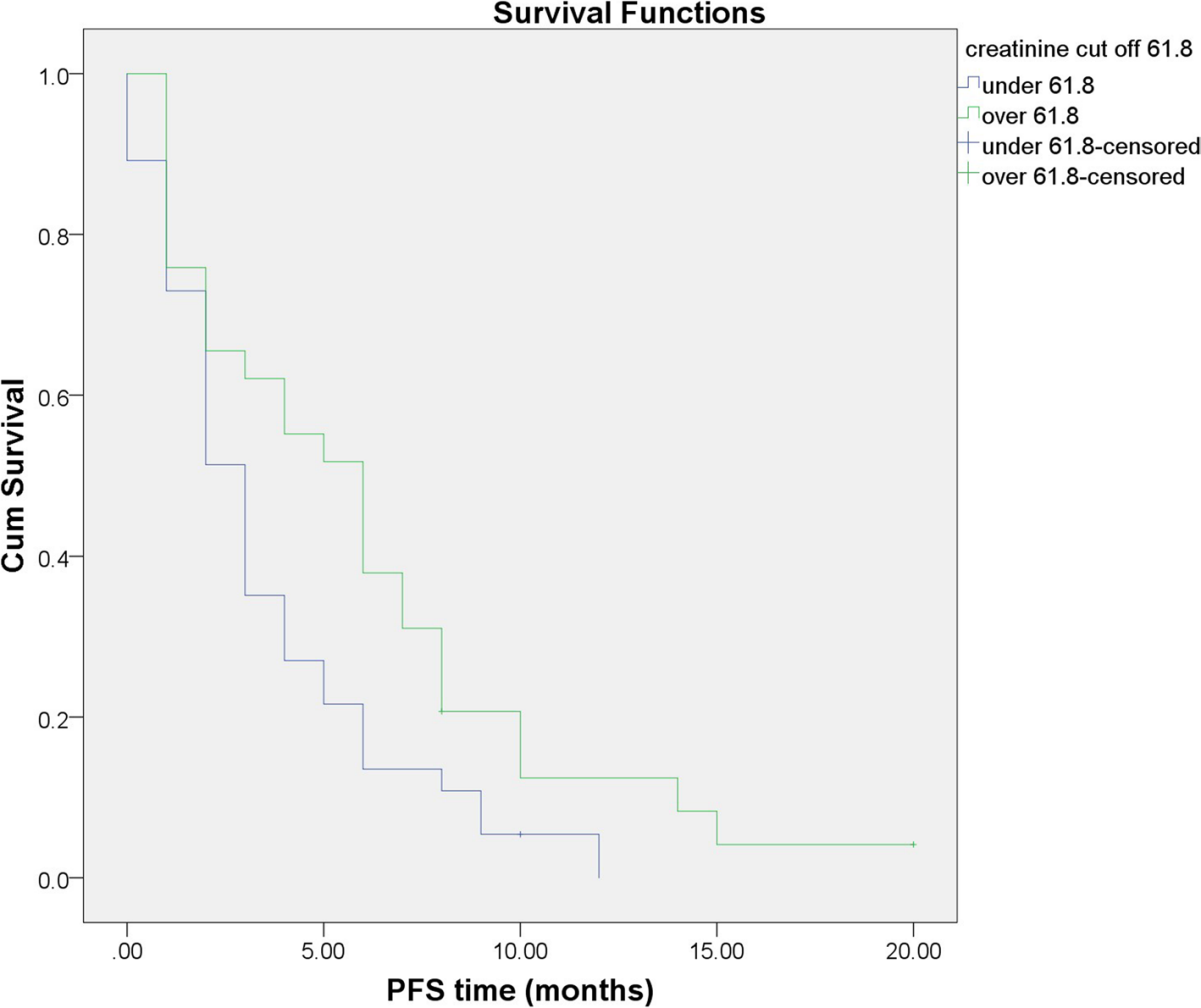
Progression-free survival in the high and low serum creatinine groups

There were also differences in PFS in patients with baseline creatinine over 61.8 μmol/L when compared with those under 61.8 μmol/L, as Fig. 3 reveals. The mean PFS in the first group was 5.99 months (95% CI: 4.181–7.806) vs. 3.51 months (95% CI: 2.501–4.526), *p* = 0.024.

### Association of serum markers with PFS time

**Fig. 4:**
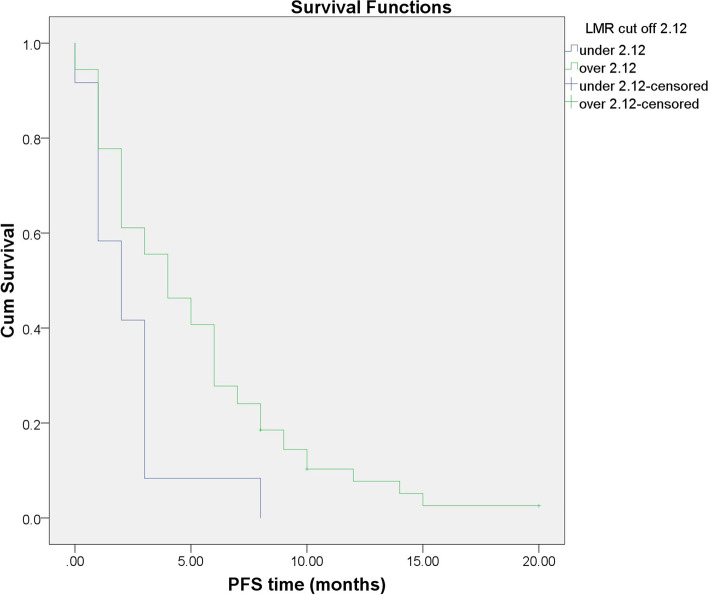
Progression-free survival in the high and low LMR groups

**Fig. 5:**
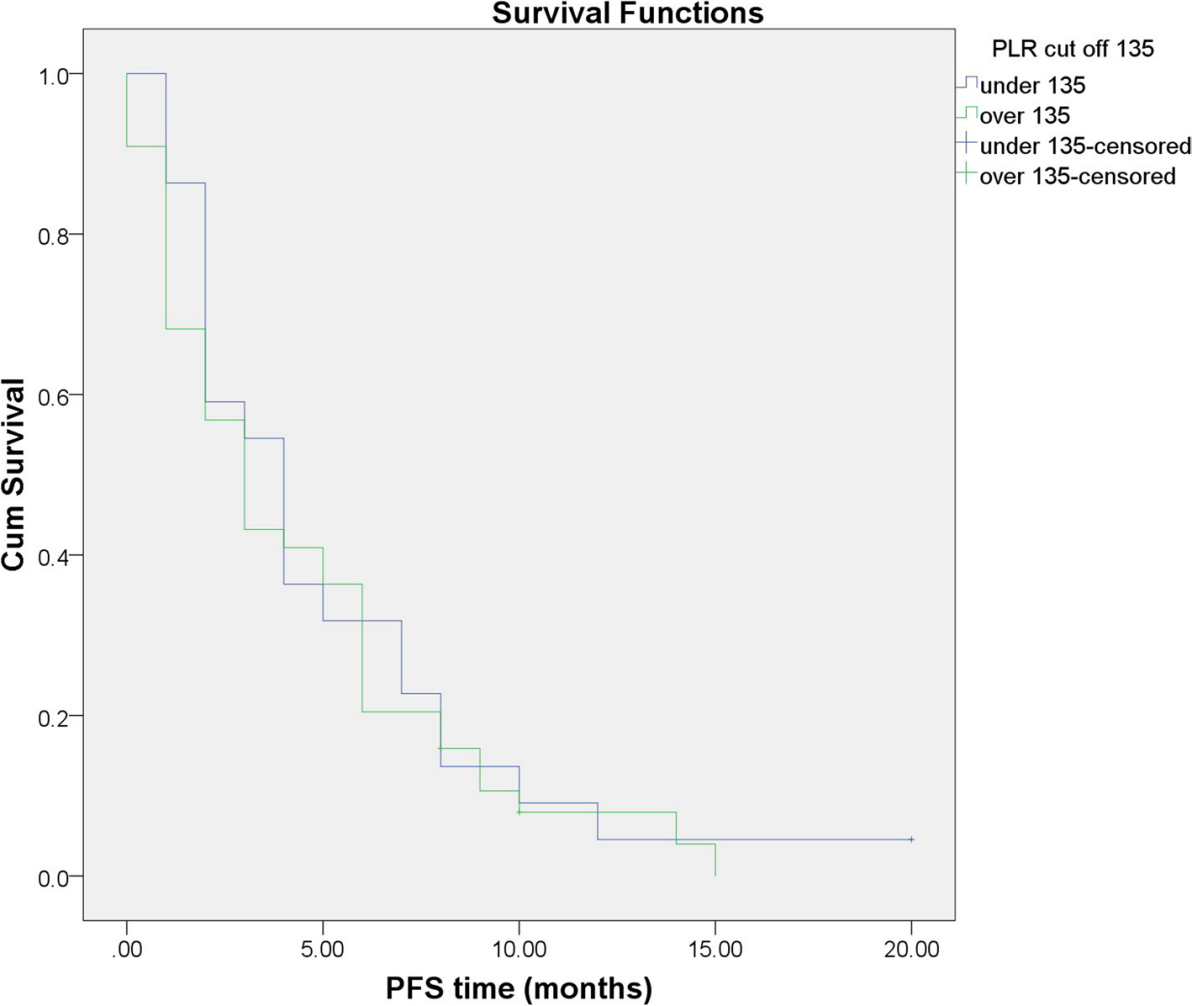
Progression-free survival in the high and low PLR groups. No significant difference was found between patients with PLR < 135 and PLR ≥ 135, *p* = 0.612

Patients with baseline LMR ≥ 2.12 had a longer PFS than those with LMR < 2.12 (mean PFS was 5.15 months [95% CI: 3.938–6.356] vs. 2.33 months [95% CI: 1.168–3.499]), *p* = 0.011 (Fig. 4). However, no significant difference was found between patients with PLR < 135 (mean PFS was 5.05 months [95% CI: 3.187–6.903]) and PLR ≥ 135 (mean PFS was 4.40 months [95% CI: 3.177–5.615]), *P* value was 0.612 (Fig. 5).

In addition, the association of each variable with PFS time, using the Kaplan–Meier method with the log-rank test, is summarized in Table 2.

**Table 2:** Kaplan–Meier method analysis of the relationship between PFS and clinical factors in patients in this study

Parameter	mean PFS (months)	95%CI		***p***-value
		Lower	Upper	
**Age (years)**				
≥ 65	4.13	2.848	5.413	0.462
<65	4.95	3.479	6.411	
**Sex**				
Female	5.66	3.656	7.66	0.178
Male	4.2	3.001	5.392	
**Treatment line**				
First line	5.02	3.525	6.509	0.556
Second or higher	4.3	2.878	5.716	
**Histology**				
Non-small cell	4.73	3.437	6.025	0.711
Small cell	4.33	2.945	5.721	
**ICIs type**				
Anti-PD-1 agent	4.74	3.463	6.01	0.695
Anti-PD-L1 agent	4.29	2.856	5.716	
**Serum ALB(g/dL)**				
< 37	1.85	0.675	3.017	<0.001
≥ 37	5.32	4.12	6.519	
**Creatinine (μmol/L)**				
< 61.8	3.51	2.501	4.526	0.024
≥ 61.8	5.99	4.181	7.806	
**BMI (kg/m2)**				
< 25	3.81	2.606	5.019	0.04
≥ 25	6.25	4.446	8.054	
**LMR**				
< 2.12	2.33	1.168	3.499	0.011
≥ 2.12	5.15	3.938	6.356	
**PLR**				
< 135	5.05	3.187	6.903	0.612
≥ 135	4.4	3.177	5.615	

### Association between PFS and clinical factors in lung cancer patients treated with ICI therapy

Subsequently, we researched the relationships between PFS and clinical factors in lung cancer patients who were treated with anti-PD-L1 or anti-PD-1 therapy. According to multivariate analyses including sex, age, histology, treatment line, ICI type, serum ALB, BMI and creatinine, the results showed that serum ALB and creatinine were independent influencing factors for PFS, while sex, age, histology, treatment line, ICI type and BMI were not significantly related to PFS (Table 3).

**Table 3:** Cox-multivariate analysis of the relationship between PFS and clinical factors in patients in this study

Parameter	Odds ratio	***p***-value	95%CI	
			Lower	Upper
**Age**	**1.009**	**0.496**	**0.984**	**1.035**
**Sex**	**0.598**	**0.094**	**0.327**	**1.091**
**Treatment line**	**1.184**	**0.559**	**0.671**	**2.088**
**Histology**	**1.154**	**0.808**	**0.364**	**3.654**
**ICIs type**	**1.08**	**0.899**	**0.329**	**3.55**
**Serum ALB**	**0.438**	**0.024**	**0.214**	**0.899**
**Creatinine**	**0.509**	**0.021**	**0.287**	**0.902**
**BMI**	**0.703**	**0.219**	**0.401**	**1.233**

## Discussion

A large retrospective study supported that melanoma patients with a BMI of 18.5–24.9 had a significantly shorter PFS than those with a BMI of 25.0–29.9 or ≥ 30 after therapy with pembrolizumab, nivolumab, or atezolizumab (median PFS: 19.9 vs. 27.2 or 28.8 months) [23]. However, one recent study showed that among 287 melanoma patients treated with ICIs, BMI was not related to clinical benefit or toxicity [13]. Another retrospective study indicated that solid malignant tumor patients, including NSCLC, melanoma, and renal cell carcinoma, with a BMI of ≥25.0 had a significantly longer PFS after ICI treatment than those with a BMI of < 25.0 (11.7 vs. 3.7 months; HR: 0.46; 95% CI: 0.39–0.54; *p* < 0.0001) [24]. Thus far, it remains to be seen whether BMI is related to clinical benefit in lung cancer patients who have received ICIs. In this study, we found that lung cancer patients with a BMI of ≥25 had a longer PFS than those with a BMI of < 25. There was a positive association with overweight and better clinical outcomes with ICIs.

Overweight and obese patients have improved survival outcomes when compared with patients with a normal body weight, which is known as the “obesity paradox” [9]. At present, the mechanism of the influence of BMI on survival outcomes after ICI therapy is just beginning to be understood. Obesity causes dysregulation of the immune response by promoting the formation of systemic meta-inflammation, which may be a potential interpretation. A recent study indicated that adipose cells in human obese subcutaneous adipose tissues could secrete a few proinflammatory cytokines and chemokines, which contribute to the establishment and maintenance of inflammation and consequently may enhance the influence on immune checkpoint inhibitors [25]. Furthermore, part of the explanation of how BMI impacts the efficacy of ICIs is that obesity increases T-cell aging, leading to higher PD-1 expression and dysfunction, or PD-1-mediated T-cell dysfunction in obesity significantly leaves tumors markedly more responsive to ICIs according to a basic experimental study [26].

Body composition is complicated, and BMI alone may not be enough to fully reflect it. BMI is not an accurate indicator of lean figures or adiposity [27]. In clinical practice, the serum albumin level is usually chosen as an indicator for patients’ nutritional status. One retrospective study pointed out that serum albumin level was not an independent predictable marker for overall response, but it was an important predictive and prognostic marker for anti-PD-1 treatment in NSCLC patients [20]. In our research, we found that the PFS of the high ALB group was much longer than that of the low ALB group, and the difference was significant. When renal function is considered, selecting serum creatinine as an indirect assessment of skeletal muscle mass represents a simple selection [16–18]. Cancers are highly proliferating and energy-demanding tissues. Especially in advanced malignant tumors, the increasing metabolic needs result in nutrient mobilization from skeletal muscle [28]. A low level of serum creatinine (< 0.7 mg/dL) is an indicator for weakness and sarcopenia, especially for older people; it is a powerful predictor of mortality in patients with chronic diseases who have a normal BMI [22]. Cachexia and sarcopenia are negative prognostic factors [14, 15]. Our results also confirmed this point, showing that the PFS time of the low creatinine group was shorter than that of the high creatinine group, and the difference was statistically significant. Low muscle mass is related to poor immunologic function because skeletal muscle provides essential nutrients for the function of lymphocytes and monocytes [29–31]^,^ which is probably related to the setting of immunotherapy based on checkpoints [32].

According to multivariate analyses including sex, age, histology, treatment line, ICI type, serum ALB, BMI and creatinine, the results showed that serum ALB and creatinine were independent influencing factors for PFS, while sex, age, histology, treatment line, ICI type and BMI were not significantly related to PFS. We suspect that the effects of BMI on PFS may be related to the levels of serum albumin and creatinine. The above results indicated that nutritional status may be an important predictor of immunotherapy for lung cancer patients. Nutrition is regarded as an important determining factor of immunoreaction, and dystrophy is the most common reason for immunodeficiency. The nutritional status of patients may influence the tumor microenvironment.

Myeloid-derived suppressor cells (MDSCs) are a marker of tumor-related inflammation and mediate the inhibition of T-cell responses in lymphoma [33]. MDSCs are viewed as a heterogeneous population of cells at different differentiation stages. MDSCs can be differentiated into polymorphonuclear and monocyte MDSCs, which are, respectively similar in morphology and phenotype to neutrophils and monocytes [34]. Studies have shown that the accumulation of monocyte MDSCs results in reduced tumor-infiltrating lymphocytes and increased tumorigenicity, aggravating immunosuppression [35]. Additionally, increasing evidence shows that there is a negative correlation between increased lymphocyte counts and tumor proliferation and invasion [36]. Platelets induce the migration of circulating cancer cells from the epithelium to the mesenchyme and promote the extravasation and metastasis of tumor cells [37, 38]. It was found that PLR was significantly connected with the appearance of irAEs in NSCLC [39]. Thus, we evaluated the influence of LMR and PLR on overall survival in lung cancer patients with ICI therapy. The results suggest that patients with baseline LMR ≥ 2.12 had a longer PFS than those with LMR < 2.12, while no significant difference was found between patients with PLR < 135 and PLR ≥ 135. The LMR could serve as a predictive biomarker for the efficacy of anti-PD-1 therapy in advanced lung cancer.

At present, one of the ‘hottest topic’ is about the complex relationship between body composition and immune reaction, and some studies have attempted to explain that point [40]. The biological basis remains indistinct, therefore further studies are needed to illustrate these mechanisms. Furthermore, we consider that in prospective randomized research with non-ICIs control arm, BMI should be regarded as a stratification factor to better define its role in the treatment of checkpoint inhibitors. At the same time, further subgroup analysis is needed to confirm whether the impact of BMI on ICIs therapeutic effect is determined by serum albumin and creatinine levels.

This study is a real-world clinical study. Of course, there are several shortcomings in this study. As a retrospective study, there is inevitable selection bias. Moreover, a total of 66 patients were enrolled in the study, and small sample sizes may lead to deviations in the results. Further prospective studies on larger queues are needed to verify these results.

## Data Availability

All data generated or analyzed during this study are included in this published article.

## Abbreviations

ICIs: immune checkpoint inhibitors
BMI: body mass index
ALB: albumin
LMR: lymphocyte to monocyte ratio
PLR: platelet-to-lymphocyte ratio
PFS: progression-free survival
OS: overall survival
NSCLC: non-small-cell lung cancer
